# Cross-border movement, economic development and malaria elimination in the Kingdom of Saudi Arabia

**DOI:** 10.1186/s12916-018-1081-z

**Published:** 2018-06-26

**Authors:** Mohammed H. Al Zahrani, Abdiasiis I. Omar, Abdelmohsin M. O. Abdoon, Ali Adam Ibrahim, Abdullah Alhogail, Mohamed Elmubarak, Yousif Eldirdiry Elamin, Mohammed A. AlHelal, Ali M. Alshahrani, Tarig M. Abdelgader, Ibrahim Saeed, Tageddin B. El Gamri, Mohammed S. Alattas, Abdu A. Dahlan, Abdullah M. Assiri, Joseph Maina, Xiao Hong Li, Robert W. Snow

**Affiliations:** 1 National Malaria Elimination Programme, Public Health Agency, Ministry of Health, Riyadh, Kingdom of Saudi Arabia; 2 Malaria Elimination Programme, Aseer Health Affairs Directorate, Abha, Kingdom of Saudi Arabia; 3 Malaria Elimination Programme, Jazan Health Affairs Directorate, Jazan, Kingdom of Saudi Arabia; 4 Directorate of Public Health, Ministry of Health, Riyadh, Kingdom of Saudi Arabia; 5 KEMRI-Wellcome Trust Research Programme, Nairobi, Kenya; 6 Malaria Elimination Unit, Global Malaria Programme, World Health Organization, Geneva, Switzerland; 7 Centre for Tropical Medicine and Global Health, Nuffield Department of Medicine, University of Oxford, Oxford, UK

**Keywords:** Saudi Arabia, Migration, Yemen, Malaria, Elimination, Conflict

## Abstract

Malaria at international borders presents particular challenges with regards to elimination. International borders share common malaria ecologies, yet neighboring countries are often at different stages of the control-to-elimination pathway. Herein, we present a case study on malaria, and its control, at the border between Saudi Arabia and Yemen. Malaria program activity reports, case data, and ancillary information have been assembled from national health information systems, archives, and other related sources. Information was analyzed as a semi-quantitative time series, between 2000 and 2017, to provide a plausibility framework to understand the possible contributions of factors related to control activities, conflict, economic development, migration, and climate. The malaria recession in the Yemeni border regions of Saudi Arabia is a likely consequence of multiple, coincidental factors, including scaled elimination activities, cross-border vector control, periods of low rainfall, and economic development. The temporal alignment of many of these factors suggests that economic development may have changed the receptivity to the extent that it mitigated against surges in vulnerability posed by imported malaria from its endemic neighbor Yemen. In many border areas of the world, malaria is likely to be sustained through a complex congruence of factors, including poverty, conflict, and migration.

**Electronic supplementary material:**

The online version of this article (10.1186/s12916-018-1081-z) contains supplementary material, which is available to authorized users.

## Background

Countries share international borders that pose specific challenges for malaria elimination and control [1]. National boundaries are political constructs without reference to the shared demographic, cultural, or social environments they bisect. People and disease vectors move between these map-drawn boundaries. Border malaria occurs because the contiguous areas share a common ecology, with frequent mixing of people, parasites, and vectors. Migrants who cross borders often represent vulnerable populations, fleeing economic hardship or civil or social disruption and may stay under the radar of official statistics [2, 3] and formal health services [4].

The Saudi Arabia–Yemen border is an area where people share a common ancestry, cultural heritage, and malaria ecology. The border divides two countries at very different stages of the pathway to malaria elimination and economic development. The border spans 1326 km from the Red Sea to the border triangle with Oman. The most densely populated area is toward the Red Sea, including Jazan and Aseer regions in Saudi Arabia, which share a 330 km land border with Yemen, and represent the last remaining foci of malaria transmission in Saudi Arabia [5, 6]. Conversely, malaria transmission in Yemeni Governorates that border Jazan and Aseer remains persistently endemic despite some progress toward control prior to 2014 [7].

This paper reviews the impacts of cross-border malaria in the last remaining territories of malaria risk in Saudi Arabia, using assembled contextual data on the potential for transmission (receptivity), local elimination strategies with an emphasis on cross-border control activities, and rates of locally acquired and imported malaria (vulnerability) in Jazan and Aseer between 2000 and 2017.

### The border

This region was once occupied by the Ottomans; however, Turkish occupation proved difficult, coming into conflict with the Zaydi Shiite Imamates in the 1880s. Following the First World War, North Yemen, the Mutawakkilite Kingdom of Yemen, was declared independent, and a provisional border treaty agreed in 1924 (Mecca Treaty) [8, 9]. During the late 1920s, the Mutawakkilite Kingdom began expanding its control along the Arabian shores moving toward Saudi Arabia as far as southern Aseer [8], bringing conflict between the two neighboring countries. This was resolved through the Treaty of Taif, signed in 1934 and establishing the boundary between the countries. The border established under this treaty represented political rather than tribal interests, but granted borderland residents the right to cross the border through certain checkpoints without restrictions, whereas other Yemeni citizens were obliged to enter Saudi Arabia with regular passports and visas [8].

**Fig. 1:**
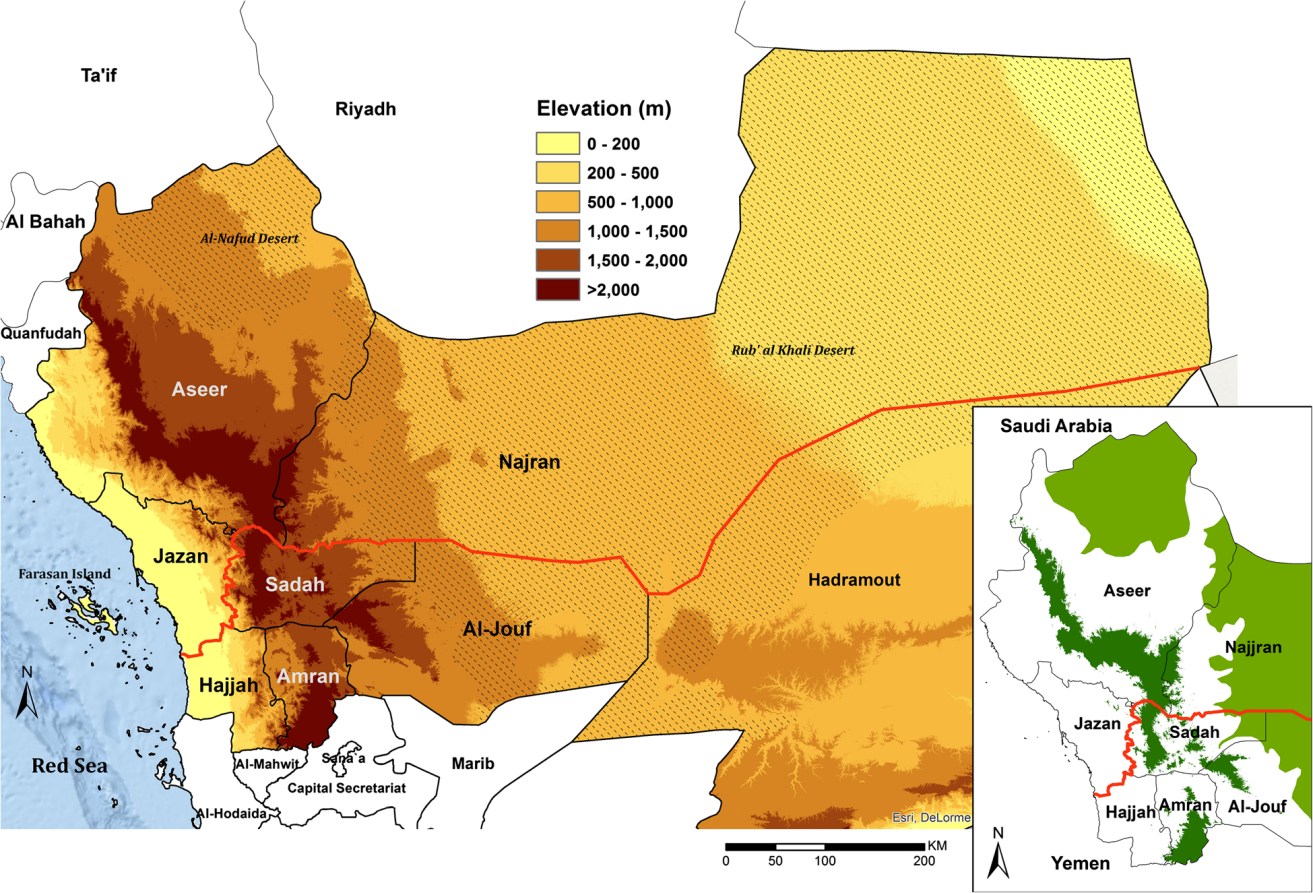
Neighboring regions/governorates along the border (red line) between Saudi Arabia and Yemen showing the desert areas (hatched areas) of Al Nafud and Rub Al Khali (the empty quarter); Insert showing margins of transmission in Jazan and Aseer regions, Saudi Arabia and Hajjar and Sadah governorates in Yemen: because of altitude greater than 2000 m (dark green) and deserts (light green)

In 1962, the Yemen Arab Republic was formed, and the remaining areas of Yemeni territory became the People’s Democratic Republic of South Yemen in 1967. Unification of the Republic of Yemen was formalized in May 1990 [9]. A renewed effort was established to resolve the border margins with Yemen, which was finalized under the June 2000, Jeddah Treaty [10] (Fig. 1). The long undefined border, previously open for borderland residents’ crossings, was now fixed by state authorities and no longer negotiable [8, 10]. Saudi Arabia began to implement a border fortification and enhanced surveillance from 2004, including the beginnings of a fenced and concrete border project [11, 12], which was completed in 2009–2010.

### Economic development and conflict

**Fig. 2:**
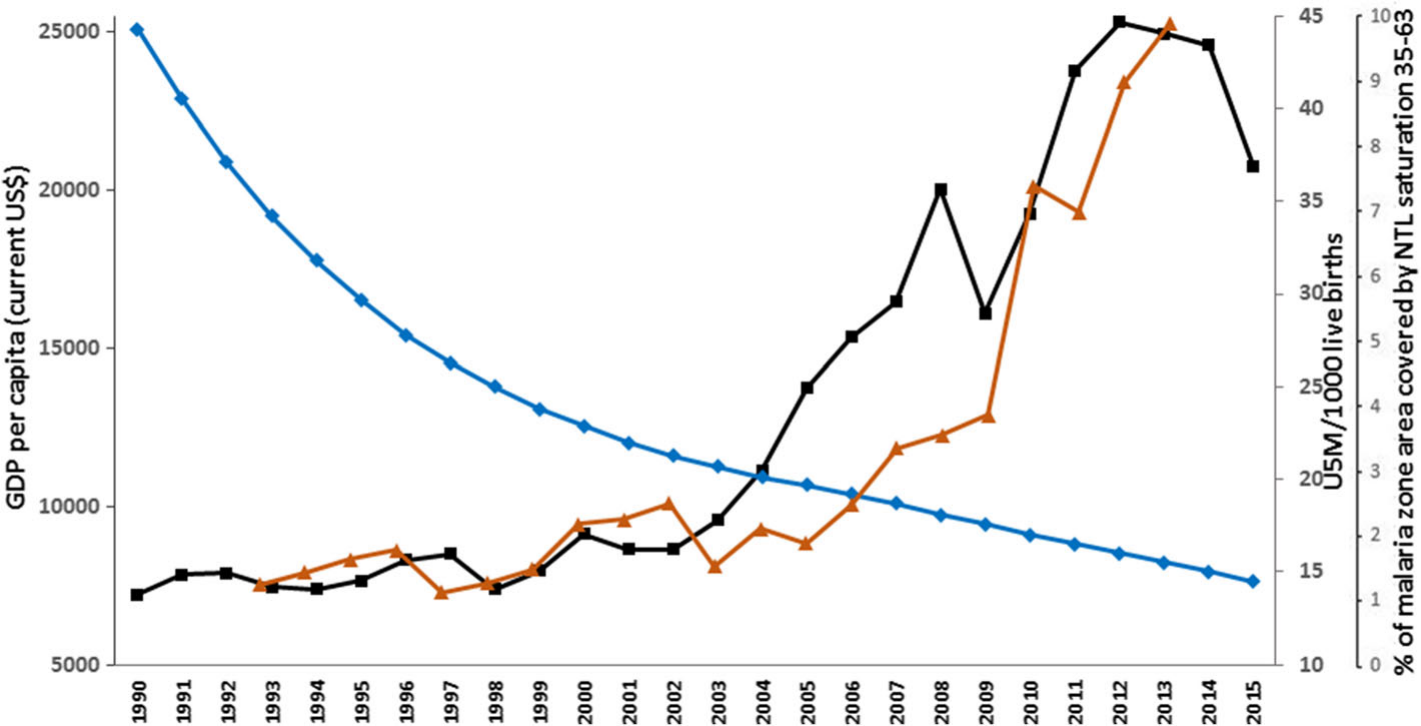
National GDP per Capita (US$) (Black line) [13], national under five mortality per 1000 live births (Blue Line) [14], and percentage of surface area where the night-time light (NTL) index is greater than 35 in the malarious areas of Jazan and Aseer regions (Brown Line). The amount of NTL, representing a qualitative scaled measure of electric light seen from space, is measured on a 0- to 63-point scale, with dense light at night measured by a measure in excess of 35 [18]. The proportion of the malaria-risk surface area of Aseer and Jazan provinces covered by intense NTL (> 35) each year between 1993 and 2013

Petroleum was discovered in 1938 in the Al-Hasa region in the east, and Saudi Arabia is now the largest oil producer and exporter. From the 1970s, wealth generated by oil revenues has been channeled by the government to increase the quality of life of most Saudis, including through urbanization, provision of primary, secondary and university education, improved healthcare systems, and new media. The GDP increased from US$ 7200 per capita in 1990 to US$ 20,730 in 2015 [13], while under five mortality fell from 44.3 to 14.5 per 1000 live births during the same period [14] (Fig. 2).

Rapid economic development has not been equally enjoyed across the Kingdom. Economic investment in the south-western regions of the country did not significantly start until after 2000. Prior to this, many areas remained rural, without access to piped water, electricity or paved roads connecting major areas of service delivery. During the 1970s, the Jazan region was largely an agricultural, rural area, dependent on ground water [15].

Annual economic and development indicators since 2000 specific to the border regions are not available; however, night-time lights, observed from earth-orbiting satellites, are a useful proxy of development and urbanization in specific areas [16–19]. The expansion of malaria risk areas of Jazan and Aseer (Fig. 1, insert), now covered by intense night-time light, suggests rapid, economic growth from 2007 (Fig. 2), coinciding with revised economic and agricultural development plans for the region, including the development of one of the largest oil refineries in Saudi Arabia, the expanded economic and naval port at Jizan, the opening of a regional University, and the start of one of the largest economic city developments in Saudi Arabia [20].

Conversely, economic and development indicators for Yemen are the worst across the Arab world [21] and investment along the border in Yemen has been almost non-existent for decades. The area has been subjected to civil disruption since the 1980s. The most recent crisis first escalated in June 2004 in the Sa’dah Governorate, when Houthi rebels came into conflict with Yemeni government forces, resulting in military clashes through to 2010, followed by a temporary cease fire in 2010 [22]. These conflicts led to a huge population migration [23]. The escalation of the civil war in Yemen began in July 2014, and further destabilized the Yemeni side of the border. The entire country is now in a humanitarian crisis of unprecedented proportions, with a complete breakdown of the economy and health systems, and more than 2 million Yemeni’s having been forcibly displaced because of the conflict [24, 25].

### Cross-border movement

Families who live along the border have close relatives living on opposite sides and there is frequent movement in both directions, following the historical migratory pattern. Since the late 1980s, there has been a rapid expansion of the agricultural sector in Jazan which has attracted informal labor from Yemen. Many illegal migrants cross the border every day, either returning the same day or staying for several days or more permanently. Following the completion of a physical barrier to cross-border movement in 2009–2010, migrants from Yemen now move either along the Red Sea from Haradh in Yemen to Altwal, Jazan, in Saudi Arabia, or make a more perilous journey through eastern mountain passes where a wall has not been built. Official statistics do not capture these illegal migrants, nor their precise number or the status of migrants after crossing the border. Since 2014, the border area has been patrolled more rigorously. However, in February 2016, migrants waiting to cross into Saudi Arabia were allowed to cross in a single humanitarian gesture, resulting in thousands of migrants entering the Jazan region through the Al Mohammed area.

### Malaria ecology

The stratification of malaria in both Saudi Arabia and Yemen has often included altitudinal and desert limits to *Plasmodium falciparum* and *P. vivax* transmission [26–28] (Fig. 1, insert). Both sides of the border, toward the Red Sea, belong to the Afro-tropical malaria ecological strata, sharing disease eco-types comparable to those of mainland Africa. The two main malaria vectors are *Anopheles arabiensis* and *An. sergentii*, with disputed and much smaller contributions from *An. culicifacies var. adenensis* and *An. d’thali* [29–31]*.*

To understand the intensity of malaria transmission prior to aggressive vector control activities in the border areas, community-based surveys undertaken before 1980 have been used [32–37]. A total of 9110 individuals were examined between 1956 and 1979 at 60 communities in Aseer and Jazan regions. Over this period, the crude *P. falciparum* prevalence rate was 6.4%, and those of *P. vivax* and *P. malariae* were 1.5% and 0.2%, respectively. Across the two bordering Governorates of Yemen (Hajjar and Sa’dah), 2075 individuals were surveyed between 1962 and 1977 at 35 communities, resulting in crude prevalence rates of 13.8% for *P. falciparum*, 0.8% for *P. vivax*, and 1.2% for *P. malariae*. *P. ovale* was not reported in any survey in either country. Overall historical transmission across the border area is probably best described as hypo-endemic, with higher rates of transmission intensity on the Yemeni side of the border compared to the Saudi side.

More recent mass blood surveys sampling 91,676 people across the Jazan region between 2012 and 2014, found only three infections (0.003%), all of which were imported from Yemen [38]. Conversely, during the 2013 Yemen Malaria Indicator Survey, 25 communities were sampled from Hajjar Governorate bordering Saudi Arabia including 2368 individuals, of whom 108 (4.6%) were found positive [39].

### Control to elimination

Over the last 50 years, efforts to control malaria in Saudi Arabia have successfully shrunk the extent of risk across the country, rendering many areas no longer receptive to imported infections [5, 6]. The eastern region was declared malaria free in 1972, despite a small outbreak of 13 *P. vivax* and 3 *P. malariae* cases reported at Ubrin, in Al Hasa region, in 1996. These were quickly contained and there has been no reported transmission in the eastern region for over 20 years. *An. superpictus* was eliminated in the northern borders with Jordan and Iraq [32] and active transmission was interrupted in the 1970s [5, 6, 40]. The hardest areas to control were located along the Red Sea, where *An. arabiensis* and *An. sergentii* sustained transmission [40, 41]. The pilgrimage routes used by those on the Hajj were protected in rural households using dichlorodiphenyltrichloroethane (DDT) for indoor residual spraying (IRS) and larviciding through the 1970s. Malaria control activities in the south-western regions of the country did not start until 1972, and control operations in Jazan and Aseer began in earnest from 1983 with expanded use of DDT for IRS and larviciding, and a special emphasis on expanding coverage of primary care to treat malaria with chloroquine.

Epidemics were reported during the mid-late 1990s in the south-western regions, coincidental with a congruence of emerging chloroquine resistance and El Nino-related flooding, and malaria case incidence began to rise [5, 6]. Renewed efforts to achieve malaria pre-elimination were launched in 2004 [5, 6, 38, 42, 43, 44], when malaria transmission was eventually constrained to Qunfudha and Al Lith Governorates of Makkah region and Aseer and Jazan regions. No locally acquired cases have been detected outside of Jazan and Aseer regions for 7 years.

### Cross-border malaria control operations

The promotion of cross-border collaboration for malaria control between Saudi Arabia and Yemen Arab Republic/Yemen began in 1979. Actual coordinated control efforts began in 1980 and included co-planned malaria control activities, IRS using DDT and mass drug administration, and an integrated approach with schistosomiasis control [36]. Meetings were held between Yemeni and Saudi ministries of health throughout the 1980s and 1990s, and included the establishment of a permanent committee consisting of joint ministries of health, interior, foreign affairs, and finance [45].

By April 2001, a Saudi–Yemeni inter-country committee was established at a meeting in Jizan, Saudi Arabia, which planned to coordinate information on malaria cases along the border, improve the mapping of focal risks, define priorities for coordinated control and increase malaria awareness among the border populations. In part, this renewed interest in a coordinated effort was a result of the first ever Rift Valley Fever epidemics on the Arabian Peninsula in 2000 [46]. In July 2001, a meeting in Sana’a, Yemen, agreed to allow accessibility of malaria technical staff of both countries to cross the borders for the purposes of malaria activities and special travel dispensation passes were provided to Yemeni and Saudi malaria program staff. This was important for joint activities that required tracing and following up of malaria cases, and demonstrated an important political intergovernmental commitment to malaria. By 2002, the cross-border joint-program of activities was established, and remained operational through to 2013. These activities were directly supported by the Saudi government and included border malaria units linked to mobile medical/surveillance teams to screen migrants with the provision of testing and treatment free of charge as well as vector control using ultra low volume fogging, IRS, and larviciding within a 10 km margin on each side of the border. From March 2015, all cross-border activities came to an end, with Saudi communities close to the Yemen border being evacuated or remaining inaccessible to the Jazan and Aseer malaria programs. All health services, including malaria control operations, collapsed on the Yemen side of the border.

### Malaria incidence in Jazan and Aseer regions

Case detection forms an integral part of all public and private health system passive disease reporting through an immediate case-notification system by phone or fax to the Directorate of Health Affairs and Vector Borne and Zoonotic Diseases Department’s reporting centres, who then investigate cases and immediate neighborhoods as part of epidemiological reporting and foci-active detection methods. In addition, historical foci of infections are investigated through active surveillance annually or through wider mass blood surveys (Additional file 1).

**Fig. 3:**
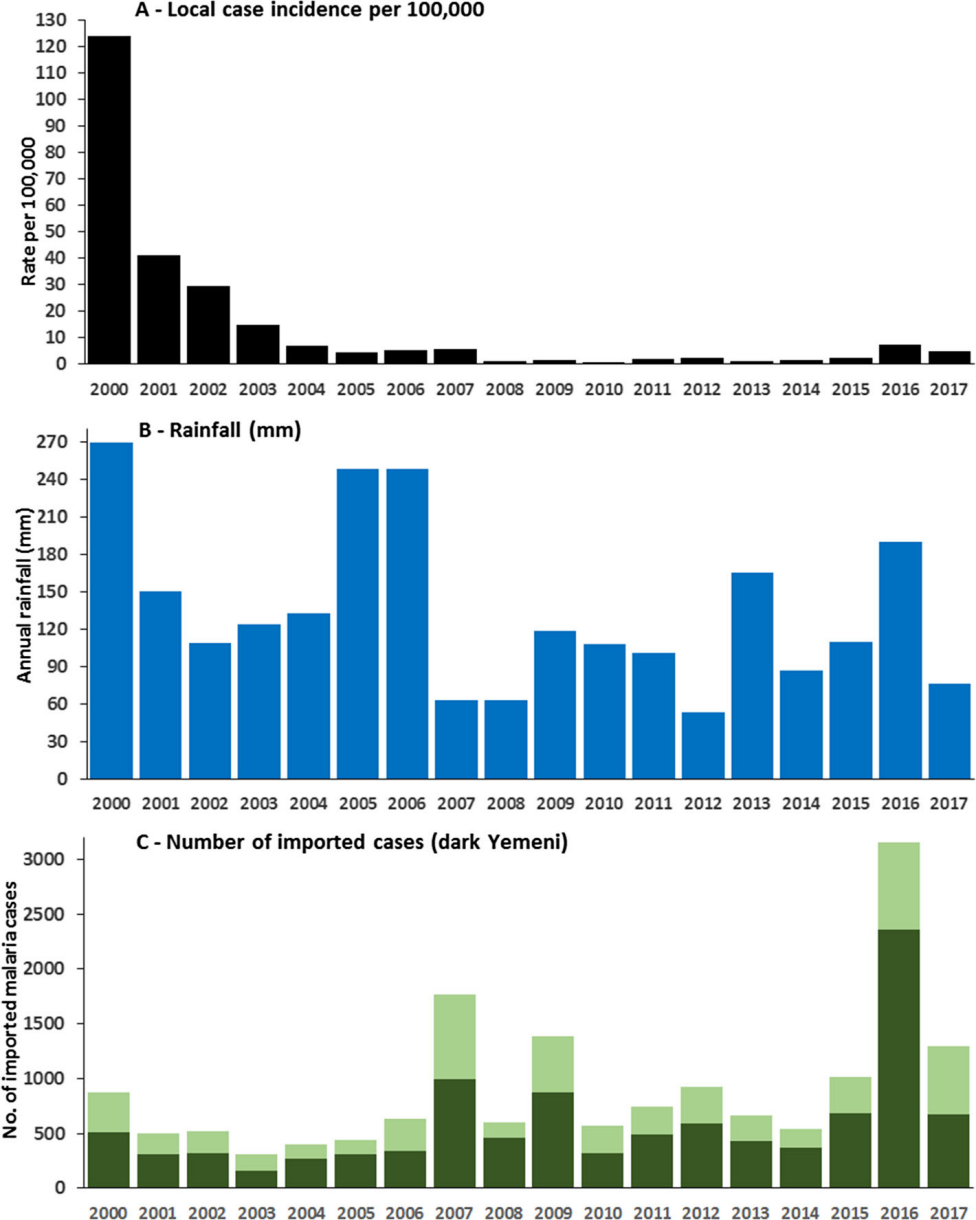
**a** Annual, locally acquired malaria case incidence in Aseer and Jazan regions 2000–2017 per 100,000 population p.a. (combined passive, active and occasional mass blood survey data). **b** Rainfall (mm) recorded at Jizan airport. **c** All imported malaria cases (bar heights), and Yemeni-origin cases (dark green), into Aseer and Jazan regions 2000–2017

Figure 3a shows the dramatic decline in the annual incidence of locally acquired malaria infections between 2000 and 2001 from 123.8 to 41.0 per 100,000 population, declining further to 7.2 per 100,000 population by 2004, when the national elimination strategy was launched. Between 2005 and 2007, following the first conflicts in Yemen and high rainfall, incidence remained between 4.7 and 5.8 per 100,000 population. From 2008 through to 2015, a period of rapid economic development in Jazan and Aseer regions (Fig. 2) and moderately low rainfall (Fig. 3b), incidence remained below 2.5 per 100,000 population per year. In 2016, a year after the escalating crisis in Yemen and moderately high rainfall, local case incidence across the two regions rose dramatically to 7.5, dropping to 4.8 per 100,000 population in 2017, levels reminiscent of 2005–2007, but still very much lower than those witnessed in the early 2000s. Overall, 99% of all locally acquired infections since 2000 were pure *P. falciparum* and all were confirmed using microscopy.

### Cross-border importation of malaria from Yemen to Saudi Arabia

Yemen has been a constant source of imported infections into Saudi Arabia given the high number of Yemeni’s seeking employment or escaping decades of civil conflict. Imported falciparum cases have been defined following epidemiological review of travel history information (Additional file 1) and are regarded as imported infections when in-migration has occurred within 5–7 days of a case being detected. Between 2015 and 2017, 32% of all imported infections detected in all regions of the country were of Yemeni origin. The number of imported malaria cases and those of Yemeni origin for the Aseer and Jazan regions are shown in Fig. 3c. Figure 3c shows the coincidence of increasing Yemeni-origin imported cases in 2007 and 2016, periods following increasing conflict in Yemen and of increased locally acquired case incidence (Fig. 3a). The increase in 2016 was largely a result of cases actively detected during a mass-screening exercise undertaken in February, when border patrols relaxed restrictions of movement of a large number of illegal migrants attempting to cross into Jazan. Of 7391 people examined during this one off mass-screening exercise, 1509 (20%) were found to be malaria positive. This migrant group included 3844 Yemenis, 3372 Ethiopians, and 175 Somalis, with infection rates of 30.2%, 8.8%, and 31.4%, respectively. All positive cases were treated with sulfadoxine-pyrimethamine-artesunate and primaquine before they continued their migration within the region.

### Understanding multiple effects on the malaria recession in Jazan and Aseer regions

From 2000 there has been a dramatic decline in locally acquired malaria in Jazan and Aseer regions (Fig. 3a). The decline observed in 2000–2004 occurred during periods of heaviest annual rainfall across the 17-year period of observation (Fig. 3b) and coincided with periods of relatively low levels of detected imported malaria (Fig. 3c). Saudi Arabia launched its elimination strategy in 2004. The Yemeni crisis, which erupted in 2006, led to a rise in imported cases from Yemen in 2007 (Fig. 3c) and a corresponding small rise in locally acquired cases (Fig. 3a). However, between 2008 and 2015, exceptionally low levels of locally acquired malaria were detected in both regions, despite a relatively constant importation of infected cases from Yemen and elsewhere. The period 2007–2013 was characterized by low rainfall and foci-directed elimination activities, changing first line treatments and increased efforts to control malaria through cross country collaboration both sides of the border. Cross-border activities came to an abrupt end due to the escalating crisis in Yemen in March 2015. Initially, physical barriers, border patrols, and a military presence prevented a large-scale exodus from Yemen into Jazan and Aseer. For a brief period in February 2016, this was relaxed and thousands of illegal migrants streamed across the border, 20% of whom were positive for malaria infection; 2016 was also a year of moderately high rainfall (Fig. 3b) and witnessed a quadrupling of locally acquired malaria cases. Local case incidence declined again in 2017 (Fig. 3b). However, it is notable that, despite the large vulnerability to renewed malaria transmission posed by massive immigration of infected illegal migrants, rates of locally acquired disease did not return to levels pre-2004. All detected, infected migrants were treated with first line therapies plus primaquine, the mass migration occurred at the end of the transmission season and 30% of all the imported cases during 2016 were detected during this migration in February.

The impact of economic development, in the face of continued threats of imported malaria, whilst hard to empirically quantify, cannot be ignored. The period of lowest malaria incidence (2008–2015) in Jazan and Aseer occurred during a time of rapid economic development (Fig. 2). It is hard to directly attribute the precise contributions of economic development versus aggressive local elimination strategies, cross-border collaboration protecting boundary areas, and drought, as these all occurred simultaneously throughout the surveillance period, and must be viewed within a plausibility framework [47]. Development affects the landscape of malaria in ways first described as bonification [48] during the malaria elimination campaigns in the United States, Palestine, Italy, and Sardinia [49–52]. It is likely that the receptivity for malaria transmission in the Aseer and Jazan regions has significantly changed over the last decade as housing has improved, paved roads and electricity connect rural areas, and urban centres have been expanded. While imported malaria continues to pose a theoretical threat to the re-establishment of malaria conditions prevalent in the early 2000s, these have not been witnessed, even following the high importation rate in 2016.

Malaria remains entrenched on the Yemeni side and conflict has led, since 2015, to a complete collapse of the health system and a humanitarian crisis that has resulted in large numbers of migrants fleeing the country or internally displaced. Those crossing the border into Saudi Arabia have been extremely difficult to enumerate, limiting any temporal denominator-adjusted epidemiological analysis. Imported infections among Yemeni immigrants (Fig. 3c) may represent only a fraction of the actual numbers. This is true of any border where migrants attempt to cross illegally, for whatever reason, and a perennial problem when studying the impact of cross-border malaria [53–55].

During 2016, it seems plausible that the large influx of an undetermined number of malaria-infected illegal migrants from Yemen might have led to the rise in locally acquired cases in the Jazan region. Equally, cross-border vector control efforts were also suspended in 2015, including areas in Saudi Arabia close to the Yemeni border. However, the likely changing receptivity combined with effective case detection and treatment through a strong health system may mean that, despite increased vulnerability through importation, the risks of re-establishing transmission in Jazan and Aseer are minimal.

In 2004, the national malaria elimination strategy’s ambition was to achieve zero malaria case incidence by 2015 [43, 56]. The problems facing elimination operations in conflict areas along the Yemeni border have meant that this target was not reached. This will continue to pose challenges for the revised elimination target of 2020 [6]; however, the continued economic investment in these regions of Saudi Arabia may change the landscape so significantly by 2020 and beyond, that malaria might be easier to eliminate during more peaceful times.

### Regional implications

In the World Health Organization’s Eastern Mediterranean Region, economic and conflict migration occur at borders between Pakistan and Afghanistan, Iran and Pakistan, Ethiopia and Sudan, and Somalia and Kenya. These settings, like the Saudi–Yemeni border, consist of neighbors at different stages of national or sub-national elimination [57]. In 2004, the Department of Communicable Disease Prevention and Control in the Eastern Mediterranean Region of the World Health Organization launched its vision for a ‘Malaria Free Arabian Peninsula’ that promoted cross-border collaboration [58, 59]. However, the success of malaria elimination is contingent on many external factors, beyond the control of regional health ambitions and national and sub-national ministries of health. The unequal balance of poverty and conflict across borders provides challenges to the neighbors who are close to elimination. These challenges need to be better articulated in regional elimination ambitions and the public health threats of forced and economic migration must be considered as part of reasoned timelines for malaria elimination. The case study presented herein provides a model to understand the complexity of elimination strategies across borders, building a historical narrative of malaria endemicity, changing receptivity through economic development and migration. This, we would argue, is a valuable exercise for all border areas where elimination is close on one side and intractable on the other.

## Conclusion

Direct attribution of the effects of imported malaria due to the sustained crisis in Yemen on locally acquired malaria in neighboring regions of Saudi Arabia is hampered by a poor description of the numbers of immigrants from Yemen. The malaria recession in the border regions of Saudi Arabia is a likely consequence of multiple, coincidental factors, including scaled elimination activities, cross-border vector control, periods of excessive and minimal rainfall, and economic development. The temporal alignment of many of these factors suggests that economic development may have changed the receptivity in Jazan and Aseer to the extent that it mitigates against surges in vulnerability posed by imported malaria from its endemic neighbor, Yemen.

## Additional file

Additional file 1: Surveillance and notification systems. (DOCX 25 kb)

## Abbreviations

DDT: dichlorodiphenyltrichloroethane
IRS: indoor residual spraying
