# Supplementary material for: Cross-border movement, economic development and malaria elimination in the Kingdom of Saudi Arabia
Source: BMC Med. 2018 Jun 26;16:98. doi: 10.1186/s12916-018-1081-z (PMC6019222; doi:10.1186/s12916-018-1081-z)

**Additional file 1: Surveillance and notification systems**

Combinations of passive and active case detection are used through networks of primary health care centres and notifications from the private sector. Cases are notified to malaria centre teams within 24 hours by facsimile, WhatsApp or a 977 SMS text messaging service. These are linked to the Ministry of Health’s Integrated Disease Notification System and Health Management Information System that feed into a web-based malaria surveillance database.

Case investigations occur within three days of notification by teams based in malaria reporting centres. Teams investigate the origins of the infection through case and travel histories, mass screening of community members within a radius of 500 m of the index case, which is maintained for a period of five weeks (reactive active case detection). All positive cases are taken to the nearest health facility by mobile teams for treatment according to the malaria drug policy of the Kingdom. In addition, entomological investigations are undertaken within the neighbourhood, followed by vector control measures to ensure 100% coverage by appropriate adult and larval stage targeted control. Details of the case and community investigations are recorded on an investigation form (Figure SI 1), which is sent by facsimile or e-mail to the malaria department in the Ministry within 72 hours of notification. This is reviewed by teams of laboratory technologists, clinicians and vector specialists to define whether the case is an imported or locally acquired infection, and a detailed report is produced.

Occasional mass blood surveys are also undertaken, either in areas of historical foci or for special investigations.

**Reporting structure**

*Immediate reporting*

After the confirmation of a positive case in any health centre, government or private hospital, a notification form is sent immediately by facsimile or telephone to the Directorate of Health Affairs and vector-borne diseases unit (malaria department) in the region. They then send an immediate facsimile to or telephone the Malaria Department in the General Department of Disease Control at the Ministry of Health. This process is intended to expedite the decision-making for any measures to tackle the disease.

*Weekly reporting*

The statistical weekly statement using a specially designed form is sent from all health centres, and government and private hospitals to the Health Affairs Directorate in all regions. After that, the directorates send the information by facsimile or email to the Head Office of National Malaria Elimination Programme in the Ministry of Health in Riyadh.

*Monthly reporting*

A specially designed form for monthly reports which includes all recorded cases during the month at health centres, and public and private hospitals, is sent from the Directorates of Health Affairs to the Malaria Department at the General Department of Disease Control at the Ministry of Health.

**Figure SI 1: case form**


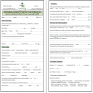

Supplement: Supplementary file 1 — Surveillance and notification systems. (DOCX 25 kb) [file 12916_2018_1081_MOESM1_ESM.docx]
